# Integration of genomic, transcriptomic and functional profiles of aggressive osteosarcomas across multiple species

**DOI:** 10.18632/oncotarget.19532

**Published:** 2017-07-25

**Authors:** Lara E. Davis, Sophia Jeng, Matthew N. Svalina, Elaine Huang, Janét Pittsenbarger, Emma L. Cantor, Noah Berlow, Bernard Seguin, Atiya Mansoor, Shannon K. McWeeney, Charles Keller

**Affiliations:** ^1^ Knight Cancer Institute, Division of Hematology and Medical Oncology, Department of Medicine, Oregon Health and Sciences University, Portland, Oregon, USA; ^2^ Department of Pediatrics, Oregon Health and Sciences University, Portland, Oregon, USA; ^3^ Division of Bioinformatics and Computational Biology, Department of Medical Informatics and Clinical Epidemiology, Oregon Health and Sciences University, Portland, Oregon, USA; ^4^ Children's Cancer Therapy Development Institute, Beaverton, Oregon, USA; ^5^ Flint Animal Cancer Center, Colorado State University, Fort Collins, Colorado, USA; ^6^ Department of Pathology, Oregon Health and Sciences University, Portland, Oregon, USA

**Keywords:** osteosarcoma, checkpoint adaptation, osterix, comparative oncology, metastases

## Abstract

In complex, highly unstable genomes such as in osteosarcoma, targeting aberrant checkpoint processes (metabolic, cell cycle or immune) may prove more successful than targeting specific kinase or growth factor signaling pathways. Here, we establish a comparative oncology approach characterizing the most lethal osteosarcomas identified in a biorepository of tumors from three different species: human, mouse and canine. We describe the development of a genetically-engineered mouse model of osteosarcoma, establishment of primary cell cultures from fatal human tumors, and a biorepository of osteosarcoma surgical specimens from pet dogs. We analyzed the DNA mutations, differential RNA expression and *in vitro* drug sensitivity from two phenotypically-distinct cohorts: tumors with a highly aggressive biology resulting in death from rapidly progressive, refractory metastatic disease, and tumors with a non-aggressive, curable phenotype. We identified ARK5 (AMPK-Related Protein Kinase 5, also referred to as NUAK Family Kinase 1) as a novel metabolic target present in all species, and independent analyses confirmed glucose metabolism as the most significantly aberrant cellular signaling pathway in a model system for highly metastatic tumors. Pathway integration analysis identified Polo Like Kinase 1 (PLK1)-mediated checkpoint adaptation as critical to the survival of a distinctly aggressive osteosarcoma. The tumor-associated macrophage cytokine *CCL18* (C-C Motif Chemokine Ligand 18) was significantly over-expressed in aggressive human osteosarcomas, and a clustering of mutations in the *BAGE* (B Melanoma Antigen) tumor antigen gene family was found. The theme of these features of high risk osteosarcoma is checkpoint adaptations, which may prove both prognostic and targetable.

## INTRODUCTION

Thirty percent of patients with osteosarcoma (OS) will die of their disease despite receiving the most aggressive treatment currently available. This survival rate has remained stagnant for nearly 30 years [1, 2]. To address this lack of progress, future research must focus on identification and characterization of these currently incurable, aggressive osteosarcomas [3, 4].

Progress in osteosarcoma research has been slow for multiple reasons, including the relative rarity of the disease. Fewer than 1,000 people are diagnosed with osteosarcoma annually in the U.S., and the majority are diagnosed as adolescents or young adults aged 10–30 [5]. This small patient population precludes rapid progress in the field by limiting the pool of candidates for clinical trials. The largest clinical trial in osteosarcoma to date enrolled patients from 19 countries – a truly remarkable collaboration – but nonetheless took over five years to complete accrual with initial results not published until four years later [6].

The genomic complexity of OS far exceeds other pediatric tumors, with a somatic mutation frequency similar to adult cancers [7, 8]. Combined with the rarity of the disease, this degree of inter- and intra- tumoral heterogeneity has stymied identification of common targetable pathways. This is exemplified by the fact that even when large scale sequencing efforts identified the PI3K/mTOR pathway as dysregulated in a significant proportion of tumors [7, 9], limited clinical annotation prevented the correlation of sequence aberrations with patient outcomes.

The “shattered” genome of osteosarcoma contains evidence of both chromothripsis and kataegis [10–13]. This degree of DNA damage suggests that checkpoint adaptation, in which cell division occurs despite irreparable DNA damage, may play a larger role in osteosarcoma than in most other pediatric cancers. Checkpoint adaptation was in fact first described in human cells using the osteosarcoma cell line U2OS, where cell cycle progression despite DNA double-strand breaks was shown to depend on PLK1 [14]. Similarly, the process through which cancer cells proliferate despite inadequate oxygen or glucose is akin to a “metabolic” checkpoint adaptation [15, 16]. In complex, highly unstable genomes such as in osteosarcoma, targeting such global checkpoint processes may prove more successful than targeting specific kinase or growth factor signaling pathways.

**Figure 1:**
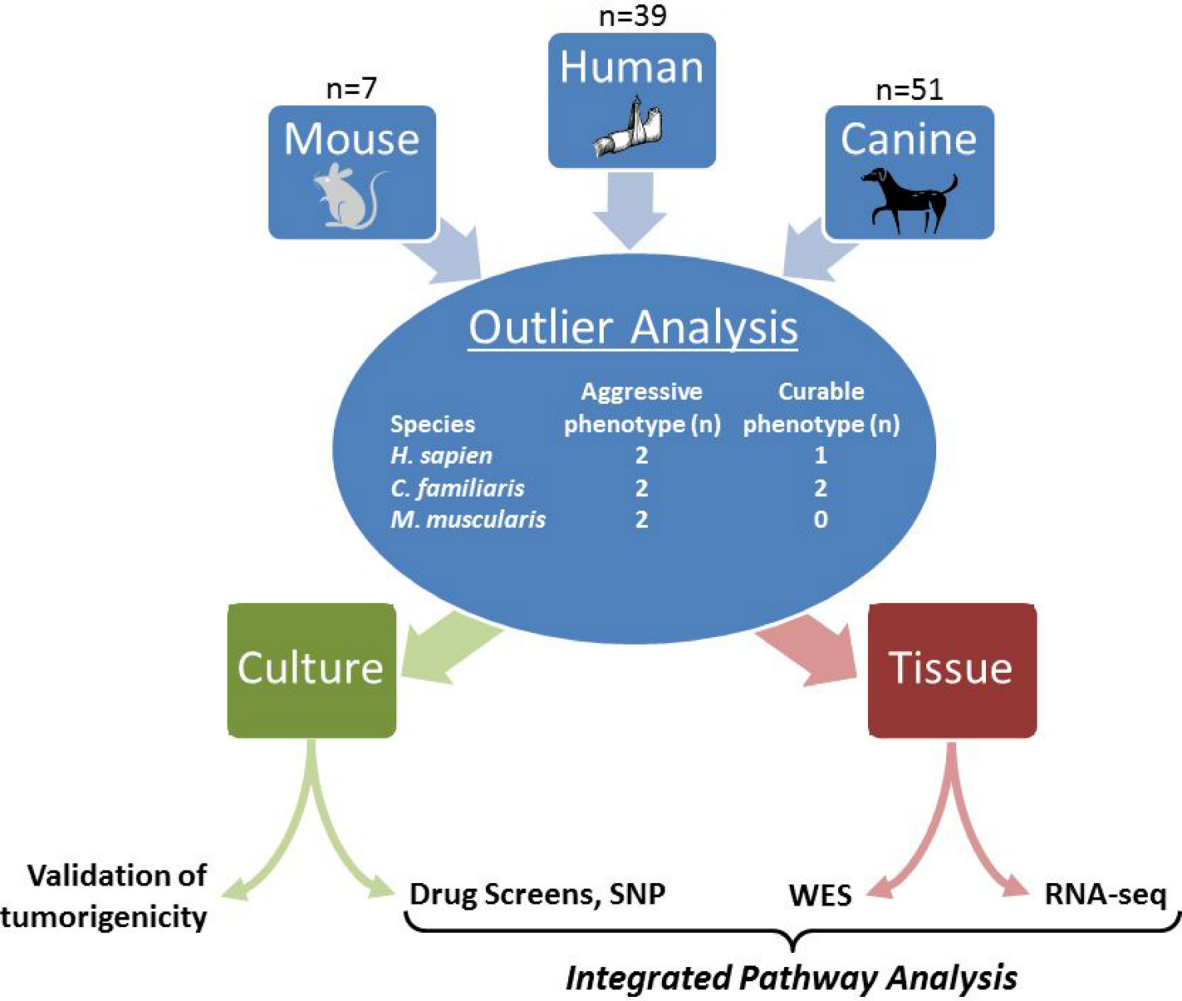
Graphical abstract

To address the slow progress in developing new treatment paradigms for metastatic osteosarcoma, we took a unique approach by focusing exclusively on lethal tumors across three species (Figure 1). With the development of a mouse model that mimics human OS and the collection of human and canine osteosarcoma tumor samples, we created a sizable osteosarcoma biorepository with extensive clinical annotation. We then selected a cohort of tumors with a particularly aggressive, metastatic phenotype, which underwent extensive functional screening and RNA and exome sequencing (Table 1). Integration of these data implicated checkpoint adaptation as a potential survival mechanism distinguishing aggressive osteosarcomas from those with a more curable phenotype.

**Table 1:** Osteosarcoma specimens included in analyses

	Specimen	Species	Months from specimen to death	Sub-specimen name	Sub-specimen description	Sequencing	Corresponding cell culture
**AGGRESSIVE**	**PCB151**	***H. sapien***	5	**PCB151**	resection	RNA, WES	
				**151JAX**	PDX explant	RNA, WES	**PCB151JAX**
				**151JAXp2X**	xenograft of PCB151JAX	RNA	
	**PCB439**	***H. sapien***	5	**PCB439**	resection (metastasis)	RNA, WES	
	**PET-10**	***C. familiaris***	3	**PET-10**	resection	RNA, WES	
				**PET-10lung**	necropsy (metastasis)	RNA, WES	
				**PET-10spine**	necropsy (metastasis)	RNA, WES	
	**PET-20**	***C. familiaris***	5	**PET-20**	resection	RNA, WES	
	**U61236**	***M. musculus***	0	**U61236**	necropsy	RNA, WES	
				**U61236liver**	necropsy (metastasis)		
				**U61236lung**	necropsy (metastasis)		**61236LUc**
	**U61323**	***M. musculus***	0	**U61323L**	necropsy	RNA, WES	**61323Lc**
				**U61323R**	necropsy	RNA, WES	
				**U61323lung**	necropsy (metastasis)		
**CURABLE**	**PCB429**	***H. sapien***	alive	**PCB429**	biopsy	RNA, WES	
	**PET-1**	***C. familiaris***	30	**PET-1**	resection	RNA	
	**PET-24**	***C. familiaris***	20	**PET-24**	resection	RNA, WES	

## RESULTS

### Genetically-engineered mice

**Figure 2:**
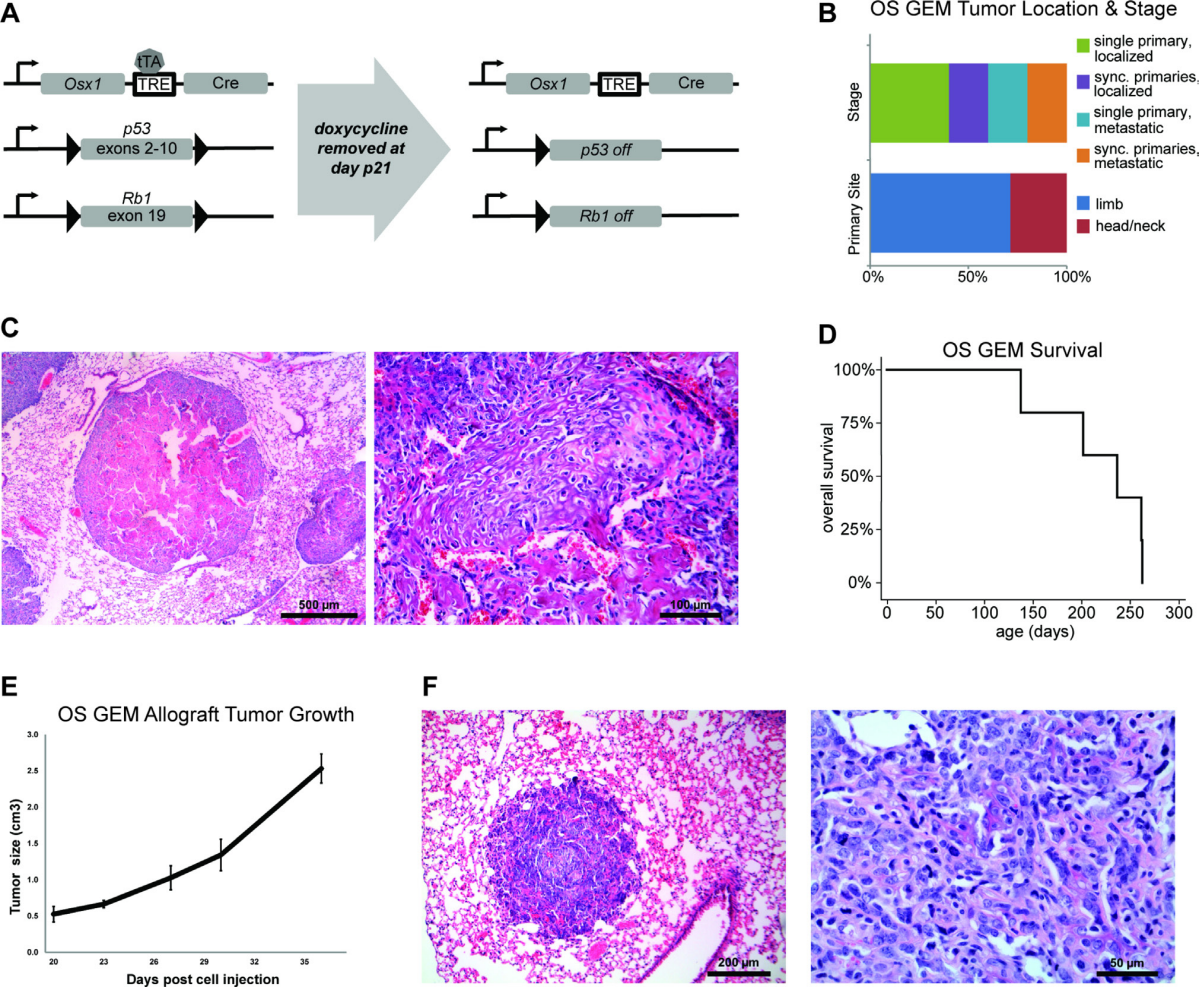
Genetically engineered mouse model of osteoblastic osteosarcoma (OS GEM) (**A**) Overview of conditional mouse model. OS GEMM pups received doxycycline through postnatal day 21. In the absence of doxycycline, Cre is expressed under the *Osx1* promoter and *p53* and *Rb1* are floxed. TRE = tetracycline-responsive element; tTA = tetracycline transactivator protein. (**B**) Distribution of site of tumor (*n* = 7 primary tumors) and whether the mice had single or multiple primary tumors and/or metastases at time of death (*n* = 5 mice). Sync. = synchronous. (**C**) H&E of OS GEM U61236 lung metastasis at low magnification (left, scale bar = 0.5mm) and higher magnification (right, scale bar = 100 μm). (**D**) Kaplan-Meier survival curve for OS GEM (*n* = 5). Median survival until tumor-specific death was 237 days. (**E**) Injection of 10^6^ OS GEM U61323L cultured cells into a hind limb results in rapid tumor development with disseminated metastases visible at time of necropsy. (**F**) H&E of one of these lung metastases at low magnification (left, scale bar = 200 μm) and higher magnification (right, scale bar = 50 μm).

Eight OS GEM (genotype *Osx1-Cre, Trp53–/–, Rb1–/–*) were generated (Figure 2A). Three OS GEM died of non-malignant causes (1 sacrificed for other purposes, 2 unknown cause but without tumor). Osteoblastic osteosarcoma developed in five OS GEM as well as two *Osx1-Cre, Trp53–/+, Rb1–/–* mice. While similar Cre:lox models have been reported previously, this model uniquely demonstrates conventional osteoblastic histology, similar to a shRNA model (Figure 2C and 2F) [17]. Tumors developed in the limbs or head/neck at an average latency time of 220 days [median 237] and metastatic and/or synchronous primary tumors were frequent (Figure 2B–2D). Ten cell cultures were established, including 7 from primary tumors and 3 from metastatic sites (Supplementary Table 2). Allografts rapidly engrafted and metastasized when these cultures were injected into immunocompromised mice (Figure 2E and 2F).

### New highly-aggressive human osteosarcoma cell line (PCB151JAX)

An 11-year-old Caucasian male developed a high-grade conventional osteosarcoma of the distal femur. He received one cycle of standard chemotherapy with vincristine, doxorubicin, cisplatin and high-dose methotrexate. He suffered a pathologic femur fracture during week 6 of treatment and underwent resection and reconstruction of his distal femur at that time. Pathologic examination of the resection specimen confirmed high-grade conventional osteosarcoma with minimal treatment response (specimen “PCB151”). Repeat chest CTs demonstrated new and enlarging pulmonary nodules that were rapidly progressive, unresectable and unresponsive to salvage chemotherapy with ifosfamide and etoposide. He died of refractory disease six months after his initial diagnosis.

**Figure 3:**
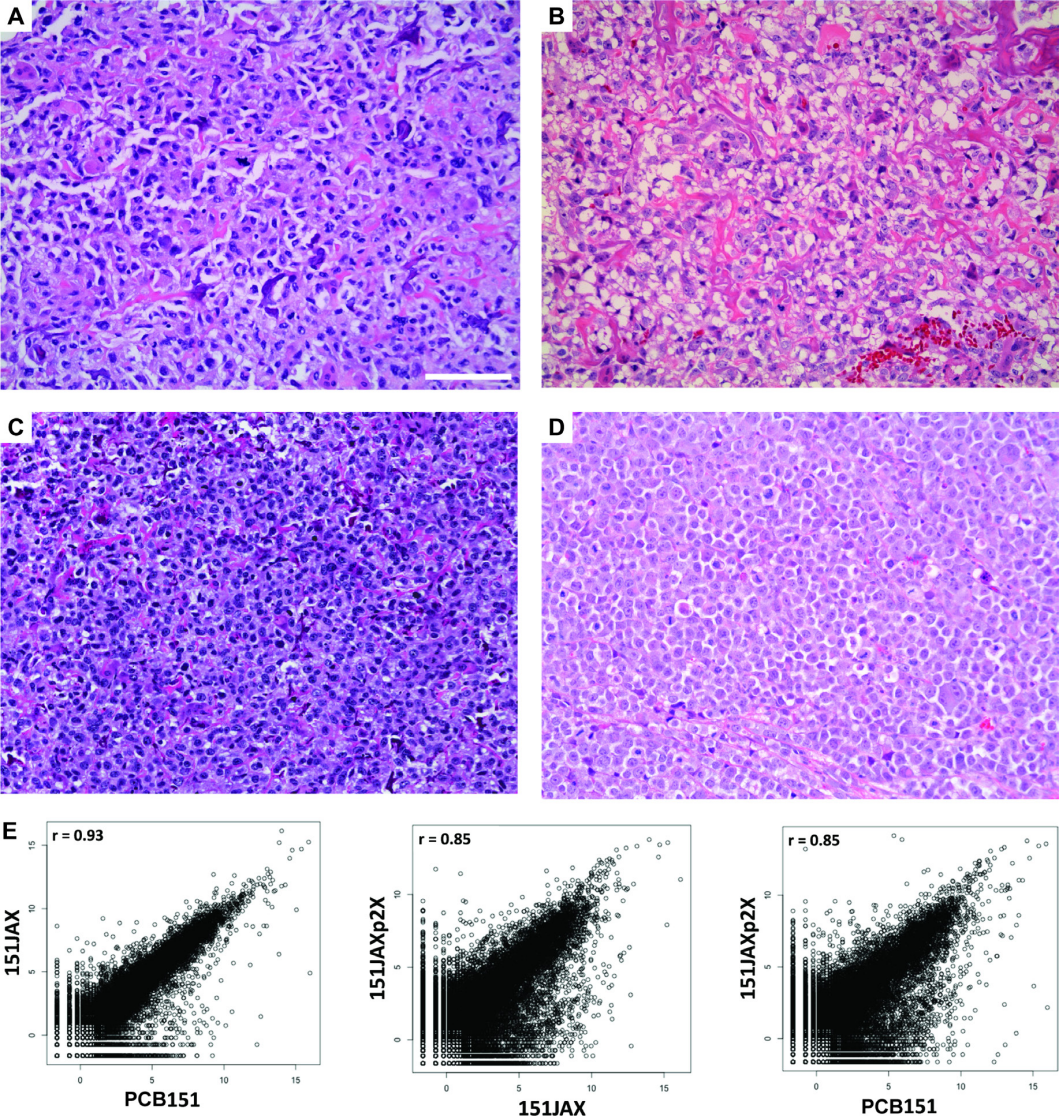
Development and validation of PCB151JAX osteosarcoma culture H&E images: (**A**) Biopsy specimen from patient. (**B**) Resection specimen from patient (“PCB151”). (**C**) Explant from PDX model (“151JAX”). (**D**) Explant from culture xenograft (“151JAXp2X”). Scale bar = 100 μm. Scatter plots (**E**) display the transcriptome correlations between the explant specimens (151JAX or 151JAXp2X) and the original patient resection specimen (PCB151). r = Pearson correlation coefficient.

A patient-derived xenograft (PDX) was established from the resection specimen PCB151. After expansion *in vivo*, the PDX explant (“151JAX”) was processed to establish a primary cell culture *in vitro* (“PCB151JAX”). After 9 weeks, the culture underwent crisis and began exponential growth in suspension. Metaphase karyotype confirmed human origin with complex abnormal related clones (Supplementary Figure 2). When injected into immunocompromised mice, PCB151JAX cells produced poorly differentiated, highly malignant tumors (“151JAXp2X”). Immunohistochemistry confirmed CD45, desmin and S-100 negativity. The progression of histology from biopsy to resection to PDX explant to culture xenograft is shown in Figure 3, with increasing cellularity and dedifferentiation over time. In the biopsy, resection, and PDX explant specimens, there are malignant cells producing eosinophilic osteoid matrix which is progressively less well developed. The 151JAXp2X culture xenograft then demonstrates a complete lack of osteoid formation consistent with anaplasia. RNA-sequencing of each of these tumors confirms a high degree of correlation between original surgical specimen PCB151 and xenograft 151JAXp2X (Figure 3E).

### Functional drug screens

The results of primary cell culture screening for sensitivity to drug panels are shown in Table 2. Drugs with IC_50_ < 1 μM against at least one cell culture are shown. IC_50_ values for these drugs are provided for cultures with fit curve R^2^ > 0.6.

**Table 2:** Cellular IC_50_ (nM) for validated primary cell cultures tested against (A) an investigator-selected drug library and (B) the PKIS compound library, as determined by a 72 hour viability screening assay

A Compound	Target(s)	AGGRESSIVE PHENOTYPE				INTERMEDIATE PHENOTYPE		
		PCB151JAX (human)	61236LUc (mouse)	61323Lc (mouse)	PCB509c human)	PCB326c (human)	PET7c (dog)	PET27c (dog)
dasatinib	ABL, SRC, KIT, PDGFR	0.02	501	1018	155	165	> 2000	906
YM155	survivin	0.37	40	150	17			
panobinostat	HDACs	10	58	97	8	53	> 2000	
nilotinib	PDGFR, KIT	26						
INK128	TORC 1/2	110	475	44	520			
carfilzomib	proteasome	112	747	687		1963		750
volasertib	PLK1	156		461				
mithramycin	SP1-4	213		371	697			
entinostat	HDAC 1/3	240	19	282	> 2000	1710		> 2000
crizotinib	ALK, MET	338	223	1008	> 2000		> 2000	1340
vandetanib	VEGFR, EGFR, RET	400	1794	788		968		
SNS-032	CDK 2/7/9	417	> 2000	1562		48	> 2000	238
JIB-04	KDM4	441	1072	182				
LY2874455	FGFR	615		647	814			
MK1775	WEE1	618	804	315				
BIX01294	histone methyltransferase	618	> 2000	1198		1621	1966	> 2000
tozasertib	AURK	638		> 2000	768			
ABT737	BCL2	664						
vorinostat	HDAC	719	928			1186	> 2000	1581
CDK1/2 inhibitor	CDK 1/2	773		> 2000	728			
ABT-236	BCL	808	1197					

**Table:** 

B Compound	Targets (partial list)	AGGRESSIVE		INTERMEDIATE	
		PCB151JAX (human)	61323Lc (mouse)	OS17 (human)	PET7c (dog)
GSK1007102B	ARK5, PDGFRA, BRSK2, PRKDs, PYK2, RSKs, MRCK	510	341	84	133
GW780056X	ARK5, PDGFRA/B, HIPK4, PRKDs, PYK2, AURKB/C, CDKs, DYRK1, GSK3	328	13	16	17
GSK579289A	ARK5, PDGFRA/B, BRSK1/2, HIPK4, KDR, NEK1/2/9, PI3K, PIM1/3	204	369		1054
GSK237701A	ARK5, PDGFRA/B, BRSK2, KDR, NEK1/2/9, PI3K, SRC, TRKB/C	461			
GW301784X	CDK2/3, TTK	47			
GW779439X	pan-kinase	867			
SB-735465	ABL, CDK6, GSK3	90			
GW778894X	ARK5, PDGFRA/B, HIPK4, PRKDs, PYK2, CDKs, GSK3, CLK, DYRK1		3		
GSK943949A	ARK5, PDGFRA/B, GSK3, PKC				482

**Figure 4:**
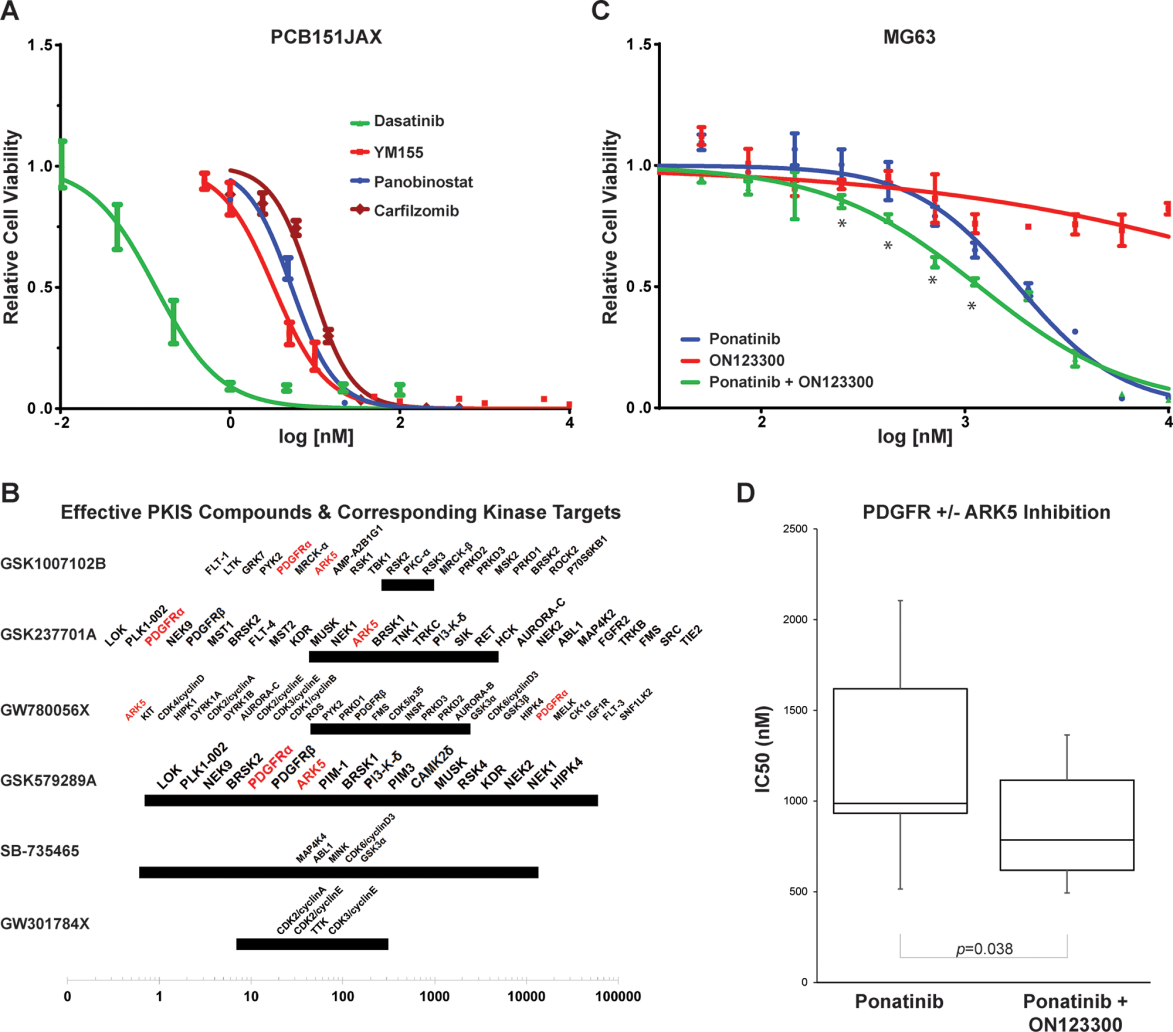
*In vitro* sensitivities of PCB151JAX and *in vitro* effect of ARK5 inhibition in osteosarcoma cell lines (**A**) Cellular IC_50_ curves from validation assays for the most effective drugs identified through screening of the investigator-selected drug panels. (**B**) Most effective PKIS compounds. The 95% confidence regions of each IC_50_ are denoted with bars; orphan kinase targets known to be inhibited within that range are noted above the bar. The most frequently recurrent targets are denoted in red. (**C**) MG63 cells do not respond to single-agent ON123300 (an ARK5 inhibitor), but synergy results when ON123300 is combined with the PDGFRα inhibitor ponatinib. Asterisks (*) denote combination index ≤ 0.7 by Chou-Talalay method (CompuSyn). (**D**) Box and whiskers plot of IC_50_ values from seven osteosarcoma cell lines: SaOS, MG63, U2OS, 143B, PCB326 and U61323L. IC_50_ for single agent ON123300 exceeded 4 μM for three lines and exceeded 10 μM for the remaining three lines tested.

PCB151JAX, a primary cell culture of aggressive human osteosarcoma, is exceptionally sensitive to the multi-kinase inhibitor dasatinib and the survivin inhibitor YM155, with IC_50_ < 1 nM on preliminary screening. Panobinostat, a pan-histone deacetylase inhibitor, has consistent *in vitro* activity as well, but we have found that this is true of nearly all cell lines regardless of histology or cell of origin. After validation at additional concentrations, more precise IC_50_ values were defined for the most efficacious compounds identified through screening (Figure 4A).

When tested against PCB151JAX, the most effective GSK PKIS compounds all inhibit ARK5 and PDGFRα (Platelet Derived Growth Factor Receptor Alpha; Table 2B and Figure 4B). BRSK2 (BR Serine/Threonine Kinase 2), HIPK4 (Homeodomain Interacting Protein Kinase 2), PDGFRβ (Platelet Derived Growth Factor Receptor Beta), PRKD1/2/3 (Protein Kinase D1, D2 and D3), and PYK2 (Protein Tyrosine Kinase 2) were also recurrent targets of effective PKIS compounds. Subsequent validation assays confirmed that inhibition of ARK5 alone is not cytotoxic, but simultaneous inhibition of PDGFRα resulted in synergy both by Chou-Talalay method and dose-effect curve shift (Figure 4C and 4D; Supplementary Figure 3).

### ARK5, a novel therapeutic target in OS

**Figure 5:**
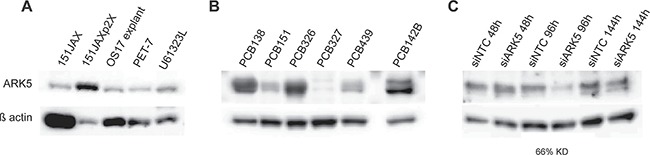
ARK5 expression is present within source tumors and is prevalent in human osteosarcomas (**A**) Immunoblotting of tumor specimens, rather than the screened cell cultures, indicates that ARK5 is expressed *in vivo*. 151JAX is the source tumor for cell culture PCB151JAX, as PET-7 is for culture PET-7c, and U61323L is for 61323Lc. 151JAXp2X is the tumor explant from culture PCB151JAX, as OS17 explant is for culture OS17. (**B**) A validation cohort of six viable human osteosarcoma tumors confirms the prevalence of ARK5 expression in an independent patient population. (**C**) Successful siRNA transfection is confirmed by reduction in total ARK5 protein by 66%, although no knockdown is seen 48 hours after transfection, suggesting a protein half-life of > 48 hours. KD = knockdown. Loading control is β actin. Images were cropped for clarity and conciseness.

Based on these results, ARK5 was identified as a novel orphan kinase with potential therapeutic implications. ARK5 is a highly-conserved AMPK-family kinase with an integral role in cellular adaptation to nutrient starvation. ARK5 activity serves as a “metabolic checkpoint” to determine cell cycle progression versus cell cycle arrest and autophagy versus apoptosis, and does so through direct interactions with AKT, LKB1 (STK11), p53 and mTOR. Western blot confirmed expression of ARK5 within the tumors that were the source tissue for primary cell cultures 61323Lc, PET7c and PCB151JAX (Figure 5A). An independent cohort of tissue fragments confirmed that ARK5 is a prevalent protein within human osteosarcomas, with variability in degree of expression (Figure 5B). siRNA knockdown of ARK5 was achieved but immunoblotting revealed a protein half-life of 96 hours (Figure 5C), limiting further utility of siRNA studies in these rapidly dividing cell cultures.

### Mutations in aggressive vs curable osteosarcomas

Whole exome sequencing of genomic DNA from tumor tissue compared to matched normal gDNA revealed a complex pattern of somatic mutations. Complete list of mutated genes can be found in Supplementary Table 3. “High impact” mutations have a high likelihood of resulting in protein disruption, while “low impact” mutations are unlikely to change protein behavior. “Modifier” mutations are usually variants within non-coding regions or within non-coding genes where impact prediction is difficult.

### Human

No high-impact mutations were identified within aggressive human tumor PCB151, most likely due to our conservative approach for mutation-calling combined with the fact that the PCB151 matched normal was obtained from paraffin-embedded tissue rather than fresh-frozen tissue, and therefore highly-degraded. Four high-impact variants were identified within aggressive human tumor PCB439, one within *NPIPB3,* one within *IPO7* and two within *ABHD2*. Notably, all human tumors contained high- or moderate- impact mutations within the largely uncharacterized gene *NPIPB3*. None of the genes with high-impact mutations found in curable PCB429 (*ARHGAP32, HTRA1, LOC388813, OSGEPL1, PTEN, TAF8, TNKS*) were mutated in either aggressive human sample. Loss of PTEN has been reported in osteosarcoma previously [13, 18].

**Figure 6:**
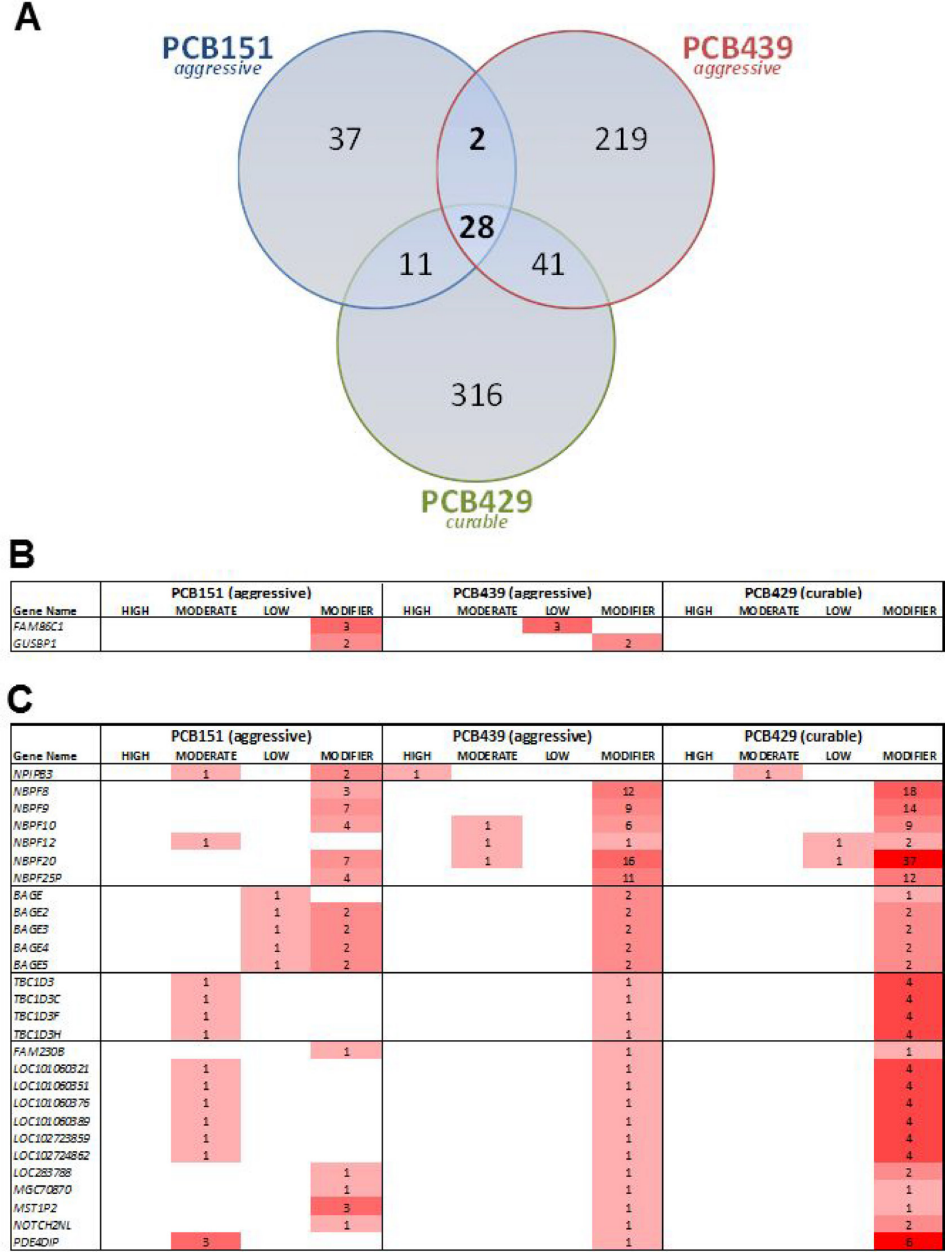
(**A**) Overlap of mutated gene in three human osteosarcomas. (**B**) Two genes were mutated in both aggressive tumors but not in the curable tumor. (**C**) An additional 28 genes were mutated in all human osteosarcoma specimens, including a high-impact mutation in *NPIPB3*, and clusters in the *NBPF*, *BAGE* and *TBC1D* families.

Only two genes were mutated in both of the aggressive human tumors but not in the curable PCB429 tumor; *FAM86C1* and *GUSBP1* contained either low- or modifier- impact mutations (Figure 6A and 6B). An additional 28 genes were mutated in all human osteosarcoma specimens (both aggressive and curable tumors, Figure 6C). When analyzing the genes that were mutated in at least two of the three human specimens, we noted a clustering of modifier mutations within the *NBPF* gene family. The neuroblastoma breakpoint family (NBPF) includes several related genes on chromosome 1. Notably, SNP array analysis of primary cell culture PCB326c revealed high level copy number amplifications of several NBPF genes, likely due to duplication of the 1q21 region. While copy number variation and translocations involving NBPF genes have been described in neuroblastoma, very little is known about their protein function [19–21]. There were also notable clusters of mutations within the *BAGE* tumor antigen family and the *TBC1D3* oncogene family. Of note, *BAGE* was significantly under-expressed (*p* = 0.02) in aggressive osteosarcomas PCB151 and PCB439 compared to the curable PCB429.

### Dog

There were no overlapping high-impact mutations between canine samples. The oncogene *JAK1* contained a high impact mutation within aggressive PET-10 tumor, while high impact mutations were identified in *CARD11*, *NF2* and *ORC1* within aggressive PET-20, and in *DPY19L2* and *SNRPC* within non-aggressive PET-24.

There were 30 mutated genes identified in all three of the samples from PET-10 (initial resection specimen and two separate metastases collected at necropsy, after systemic therapy), suggesting these variants occurred early in oncogenesis. *EML5*, encoding a microtubule protein, contained multiple moderate-impact mutations in all PET-10 samples.

*CORTBP2* and *LAMA2* contained multiple modifier mutations in both aggressive tumors but were not mutated in non-aggressive PET-24. *ASUN*, *CACNA2D1*, *TSPEAR*, and *PLCG2* were also only mutated in the aggressive-phenotype samples.

### Mouse

There were no overlapping high-impact mutations between mouse samples. Two genes, *DNAH7A* and *EIF4ENIF1*, were mutated in all three aggressive *Osx1-Cre, Trp53–/–, Rb1–/–* mouse tumors.

*BARX2*, *FLNA*, *HHEX*, *LOXHD1*, *PPP2R5A*, and *SON* contained high-impact mutations in U61236. *PPP2R5A*, which is involved in the negative control of cell growth, was also mutated in U61323R (moderate-impact variant). Loss of PPP2R5A function predicts tumor sensitivity to PARP inhibition due to impaired homologous recombination [22]. High-impact variants of *IGSF10* and *MYO9A* were identified in U61323L only. *CRABP2*, *MOB3A*, *OTOG*, and *STAB2* contained high-impact mutations in U61323R. No other species contained mutations in these genes.

### Differential gene expression of aggressive vs curable osteosarcomas

First, we compared differential gene expression (DE) between aggressive human osteosarcomas (PCB151 and PCB439) and curable osteosarcoma (PCB429). We also compared expression with that of pooled RNA from normal bone. Similar analyses were undertaken comparing aggressive canine osteosarcomas (PET-10 and PET-20) and non-aggressive canine osteosarcomas (PET-1 and PET-24) as well as aggressive canine osteosarcomas versus normal cultured canine osteoblasts (CnOB). All mouse specimens had an aggressive phenotype, therefore comparison was with pooled RNA from normal mouse bone only. Complete DE gene list in Supplementary Table 4.

### Human

In the human tumor cohort, no genes remained after False Discovery Rate (FDR) adjustment for multiple testing, therefore unadjusted *p*-values less than 0.05 were considered significant. Two genes were over-expressed in the aggressive human specimens compared with the non-aggressive tumor, *CCL18* (*p* = 0.03) and *HTRA3* (*p* = 0.04). *HTRA3* is a candidate tumor suppressor and the target of therapeutics currently in development [23, 24]. *CCL18* is a macrophage cytokine implicated in promoting cancer metastasis [25].

### Dog

Four genes were differentially expressed (adjusted *p* < 0.05) between the aggressive canine specimens compared with the non-aggressive tumors: *MFAP4,* *CHRDL1*, *LOC100684002*, and *TMSB4X*. *TMSB4X*, which encodes an actin sequestering protein, was also significantly under-expressed in mouse OS. Compared to normal canine osteoblasts, six genes were significantly (adjusted *p* < 0.05) under-expressed in the aggressive tumors: *GDNF,* *CEMIP* (*KIAA1199*), *GDF6*, *ALPK2*, *GREM1*, and *DHRS2*. *CEMIP* is a target gene of the Wnt/β-catenin signaling pathway and is known to promote cancer cell migration [26–28]; it is thus surprising that all four canine osteosarcoma specimens – aggressive and non-aggressive – demonstrated marked and significant under-expression compared to normal osteoblasts. The kinase ALPK2 is also reported to be under-expressed in gastric cancer and may be downregulated by oncogenic KRAS [29–31]. No other species contained differential expression of these genes except as noted.

### Mouse

850 genes from the *Osx1-Cre, Trp53–/–, Rb1–/–* mouse tumors were differentially expressed as compared to normal mouse bone (adjusted *p* < 0.05), 814 down-regulated and 36 up-regulated. *Cdkn2a* was dramatically over-expressed (adjusted *p* < 0.001) in the OS GEM tumors compared to normal bone, consistent with ineffectual cell cycle control in the absence of p53 and pRb.

### Integration of functional, genomic & transcriptomic data

Beyond the phenotype outlier analysis (aggressive versus curable), we also undertook a precision-medicine approach to integrating all data types for specific tumors. The individual tumor results of whole exome sequencing (WES) and RNA sequencing (RNA-seq) for aggressive human sample PCB151 were integrated with PCB151JAX drug screen results. Similarly, sequencing data for aggressive OS GEM sample U61323L were integrated with the U61323Lc drug screen results. For each individual, REACTOME-curated cellular pathways were systematically prioritized based on the presence of DNA mutations and differential RNA expression, and filtered to further prioritize genes encoding known proteins targets of drugs that were effective in the screen.

### PCB151

**Figure 7:**
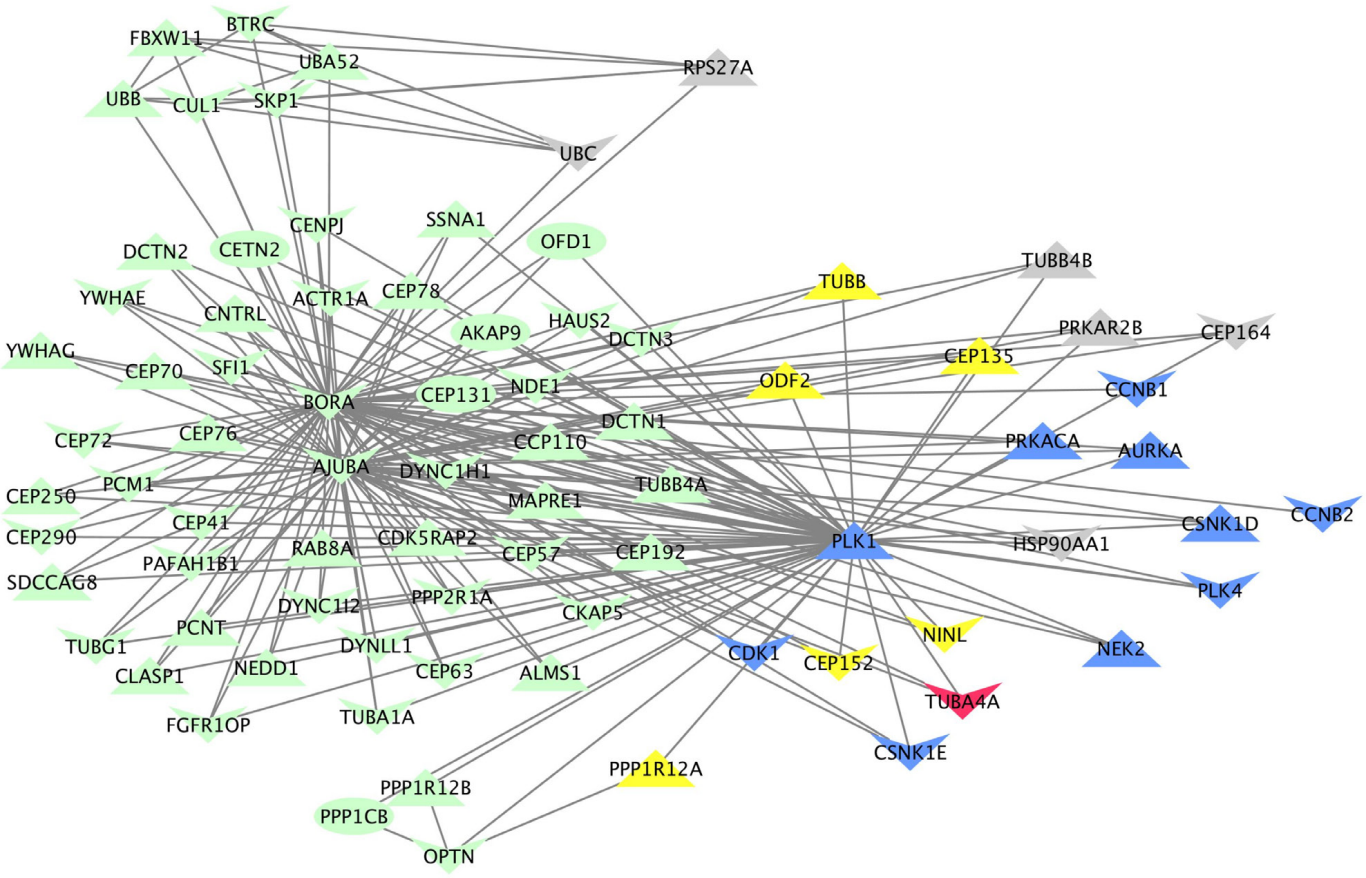
Visual representation of the “Regulation of PLK1 activity at G2/M transition” REACTOME pathway, annotated with genomic, transcriptomic and functional data specific to patient PCB151 Each of the 78 genes in the pathway are represented as nodes and known interactions are depicted by arrows. Node shape indicates the direction of copy number variation (down = deletion, up = amplification). Blue nodes are targets of drugs with *in vitro* efficacy. Grey nodes are genes with aberrant expression (fold-change greater than 2 but not statistically significant). Yellow nodes are differentially under-expressed genes (*p* < 0.05). The red node (*TUBA4A*) contains four moderate-impact mutations.

The complete list of significantly over-represented pathways (unadjusted *p* < 0.05) is in Supplementary Table 5. After integrating WES, RNAseq and drug screen data, the top ranked pathway for tumor PCB151 was “Regulation of PLK1 Activity at G2/M Transition” (unadjusted *p*-value = 0.003), illustrated in Figure 7. Drug targets are primary or secondary hubs in this pathway, and interact with many DE and mutated genes.

### U61323L

The complete list of significantly over-represented pathways (unadjusted *p* < 0.05) is in Supplementary Table 5. After integrating WES, RNAseq and drug screen data, the top ranked pathway for tumor U61323L was “Glucose Metabolism” (unadjusted *p*-value < 0.001). This was an unexpected result, but is consistent with the identification of ARK5 as a potential therapeutic target through earlier functional screens. REACTOME does not include ARK5 within the members of the Glucose Metabolism Pathway, therefore this is an independent supportive finding of glucose metabolism as a therapeutic target in osteosarcoma, potentially through ARK5 inhibition.

## DISCUSSION

Death from metastatic disease remains the reality for nearly 30% of all patients diagnosed with osteosarcoma. Clinicians have only palliative cytotoxic chemotherapy or morbid metastectomies available for the treatment of patients with metastatic osteosarcoma, and clinical trials for advanced OS remain few and far between. Although rare, osteosarcoma most often strikes young people, with decades of potential life-years ahead of them. Our field has been called to action with good reason.

For years, we have recognized the utility of studying osteosarcomas that spontaneously arise in dogs. More recently, genetically-engineered mouse models of osteosarcoma have been developed. In an effort to facilitate more rapidly translatable research, we gathered these preclinical tools together to take a comparative oncology approach to metastatic osteosarcoma. This report highlights the advantages and limitations of this approach while identifying potential therapeutic targets for future evaluation.

The extensive clinical annotation of our biorepository allowed for identification of phenotypic extreme outliers, which are by definition limited in number. The genomic and transcriptomic data from these outliers (“aggressive” versus “curable”) should be hypothesis-generating. The small sample size and limited sequencing depth hinders our ability to draw firm conclusions from this dataset, but these same factors may lend extra credence to findings that reach statistical significance nonetheless.

Perhaps the greatest challenge for this type of analysis is the genome annotation for *Canis familiaris*. The *Homo sapiens* genome assembly Hs19 includes 82,960 gene variants and Mm10 (*Mus musculus*) includes 59,121 gene variants, while CanFam3 includes only 29,884. The poor annotation for the dog genome limits our ability to perform many desirable cross-species analyses.

Despite these limitations, several noteworthy findings emerged. First, glucose metabolism is a relatively unexplored pathway in osteosarcoma pathogenesis but may be worth pursuing. Glucose metabolism was the most significant REACTOME pathway identified through a precision medicine approach to the OS GEM tumor U61323L. In addition, through separate and independent analyses of functional assays, ARK5 was identified as a recurrent target of the most effective PKIS compounds against OS cells from all three species. Importantly, we showed that this is not simply an *in vitro* phenomenon, but that ARK5 is expressed within osteosarcoma tumor tissue. During glucose starvation, ARK5 is phosphorylated by AKT, which leads to evasion of apoptosis [32]. Altered energy metabolism in osteosarcoma cells may produce a dependence on an ARK5-mediated cell survival mechanism. Interestingly, ARK5 overexpression has been associated with increased tumor cell invasion and metastatic potential in gliomas and breast cancer [33, 34]. ARK5 was significantly over-expressed in the highly metastatic OS GEM tumors in our analyses (*p* = 0.04).

We also uncovered a significant over-expression of *CCL18* in both aggressive human osteosarcomas that was not present in the curable human tumor. CCL18 is a cytokine released from tumor-associated macrophages that binds to CCR8 and has been implicated in the epithelial-mesenchymal transition of breast, pancreatic and lung cancers [35–37]. CCL18 expression by tumor-associated macrophages was recently shown to be essential for cancer metastasis in a humanized mouse model of breast cancer [25]. A clustering of mutations in the *BAGE* gene family, coding for tumor antigens recognized by cytotoxic T-cells, also implicates a role for immune evasion in osteosarcoma. The *BAGE*-family mutations and over-expression of *CCL18* is intriguing. Although we cannot draw conclusions based on two samples alone, these findings are worth further investigation when considering the known utility of immune modulators in metastatic osteosarcoma. For example, when muramyl tripeptide phosphatidyl-enthanolamine (MTP-PE) is used to stimulate macrophage cytotoxicity, patients with osteosarcoma have improved survival [2]. Similarly, inhaled granulocyte-macrophage colony stimulating factor has been used in an attempt to stimulate an immune response to OS pulmonary metastases, which often evade immune mechanisms by elimination of surface markers such as Fas [38, 39].

Finally, using a precision medicine approach to integrate functional, transcriptional and mutation data for the exceptionally aggressive osteosarcoma PCB151JAX, we identified the PLK1 pathway as the most aberrant pathway with potential for therapeutic targeting. Progression through the cell cycle requires PLK1-mediated degradation of WEE1, which increases CDK1 activity and promotes M-phase [40]. PLK1 activity is also essential for mitotic exit [14, 40]. Through our integrated analysis, we have evidence that PLK1-mediated checkpoint adaptation may be critical to the survival of PCB151JAX. Checkpoint adaptation, in which cell division occurs despite irreparable DNA damage, is an important mechanism of oncogenesis and cancer growth, and interestingly was first described in human cells in the osteosarcoma cell line U2OS [14]. Targeting checkpoint adaptation in osteosarcoma has not been pursued to date clinically, but may be feasible now that PLK1 inhibitors are entering clinical trials.

Together, these data indicate that in complex, highly unstable genomes such as in osteosarcoma, targeting aberrant checkpoint processes may prove more successful than targeting specific kinase or growth factor signaling pathways. Given the limited therapeutic options and dismal prognosis for patients with metastatic osteosarcoma, targeting aberrant metabolism or cell cycle checkpoints as part of the treatment regimens for these patients is certainly worth further investigation.

## MATERIALS AND METHODS

### Genetically-engineered mice

*Osterix-Cre* and conditional *Rb1* and *p53* lines have been previously described [41–43]. We crossed these animals to generate compound conditional *Osx1-Cre*, homozygous *p53*, homozygous *Rb1* genetically engineered mice (GEM), hereby referred to as OS GEM mice. Genotyping detailed in Supplementary Methods. Construct depicted in Figure 2A. Animals were maintained on doxycycline 0.2 mg/mL in drinking water from conception to postnatal day 21. Animals were sacrificed once tumors reached 2 cm^3^ or, particularly in the case of facial tumors, when humanely indicated.

### Primary cell cultures and tumor tissue

Primary cell cultures were established as described in Supplementary Methods and as described previously [44]. Clinical details of all human and canine patients are also included in Supplementary Methods.

### Confirmation of primary cell culture tumorigenicity

Xenografts from multiple primary cell cultures from humans (PCB151JAX, OS17) and dogs (PET7c, PET27c) successfully engrafted and produced osteosarcoma tumors, as did allografts of 61236LUc (U61236 lung metastasis culture) and 61323Lc (U61323 left limb culture). *In vivo* procedures are detailed in Supplementary Methods.

Due to the exceptionally high rate of DNA aberrancies within osteosarcomas, copy number variation analysis was used to confirm the predominance of cancer cells within additional primary cell cultures from human tumor specimens. GISTIC 2.0 analysis resulted in a call rate distribution in three “clusters” (Supplementary Figure 1): one with a distribution similar to normal samples, one distinct from the normal sample call rate distribution (highly aberrant and consistent with cancer), and a third indeterminate group with a call rate distribution distinct from both the normal and cancer call rate distributions, likely representing a mixed population of cells. This method validated human osteosarcoma primary cell cultures PCB509c and PCB326c as containing predominately cancer cells.

### Identification of two phenotypically-distinct cohorts of osteosarcoma

From our biorepository of clinically-annotated osteosarcoma specimens, tumors with an exceptionally aggressive clinical course were identified for further study (Table 1). One human tumor with favorable clinical prognostic factors (appendicular primary tumor site and > 90% necrosis following neoadjuvant chemotherapy) and two canine tumors from dogs that survived > 1 year after diagnosis were identified as non-aggressive comparators. All OS GEM tumors were considered aggressive; the two that were included in the following analyses were associated with gross metastatic disease at necropsy.

### Clinical/pre-clinical drug & compound screens

Cultured cells were screened for sensitivity against a panel of 105 investigator-selected drugs (Supplementary Table 1) and the Published Kinase Inhibitor Set (PKIS) [45]. The majority of the investigator selected drugs are in clinical trials, while the remainder are either FDA-approved drugs or preclinical tool compounds. The PKIS, which includes 402 kinase inhibitors with corresponding inhibition profiles against 224 kinases, was provided by GlaxoSmithKline [45–47]. Each drug or PKIS compound was plated in four concentrations in triplicate in 384-well format and incubated for three days. In addition, a highly specific ARK5 (NUAK1) inhibitor, HTH-01-015, kindly provided by Dr. Nathanael S. Gray [48], was also evaluated. Cell viability was determined by CellTiterGlo luminescent assay (Promega #G7572). Effective drugs identified through screening were validated at additional concentrations. Using Prism 6 (GraphPad Software Inc.), these data were transformed logarithmically to fit four parameter nonlinear logistic curves and determine IC_50_ values.

### DNA and RNA sequencing

RNA was isolated from tissue preserved in TRIzol reagent (Invitrogen #15596). DNA was isolated from fresh frozen tissue or peripheral blood mononuclear cells, with the exception of the matched normal tissue for PCB151, which was formalin-fixed, paraffin-embedded. Whole exome sequencing and RNA sequencing (both paired-end, 100 bases, indexed) was completed with an Illumina HiSeq 2000 Sequencer. Data pre-processing included inspection of FASTQC QA/QC metrics (http://www.bioinformatics.babraham.ac.uk/projects/fastqc/) prior to alignment with Subread [49] to the appropriate species build (UCSC hg19 for human, UCSC mm10 for mouse, Ensembl CanFam3 for dog).

Putative differential expression analysis was conducted by fitting a linear model to each gene/transcript using edgeR [50, 51]. Using a conservative approach for identifying significance, we required both the common dispersion analysis and tagwise dispersion analysis False Discovery Rate (FDR) adjusted *p*-values to be less than 0.05.

Somatic mutation calls were made with MuTECT following the GATK Best Practices from the Broad Institute [52]. Using only mutations rated as high confidence, we used SnpEff to annotate the variants’ impact as high, moderate, low or modifier [53]. Note, a gene may have more than one mutation and thus may have mutations of multiple different impacts.

### Integration of functional, genomic & transcriptomic data

Pathway enrichment analysis was conducted based on patient-specific DNA mutations and species-specific differential RNA expression (compared to normal bone). Pathways were prioritized based on unadjusted *p*-value < 0.05 and filtered for pathways that included at least one target of an effective drug. Drug targets were determined from the investigator-selected and PKIS compound screens by correlating effective drugs with their known protein targets. Mappings between UCSC Gene ID to Uniprot ID were obtained via UCSC and Uniprot to REACTOME mappings was obtained through REACTOME. All analyses were conducted at the UCSC Gene ID level.

## SUPPLEMENTARY MATERIALS FIGURES AND TABLES










