# Supplementary material for: Integration of genomic, transcriptomic and functional profiles of aggressive osteosarcomas across multiple species
Source: Oncotarget. 2017 Jul 25;8(44):76241–56. doi: 10.18632/oncotarget.19532 (PMC5652702; doi:10.18632/oncotarget.19532)
Supplement: Supplementary file 1 [file oncotarget-08-76241-s001.pdf]

Integration of genomic, transcriptomic and functional profiles of aggressive osteosarcomas across multiple species

SUPPLEMENTARY MATERIALS

MATERIALS AND METHODS

OS GEM

Rb1 and *Trp53* strains were obtained from the NCI Mouse Repository. Both the *Rb1* floxed strain (#01XC1) and the *p53* floxed strain (#01XC2) were deposited by Dr. Anton Berns. The *Osterix-Cre* floxed strain (#006361) was purchased from The Jackson Laboratory (Bar Harbor, MA, USA) and deposited by Dr. Andrew McMahon.

Genotyping

The structure of targeted alleles is shown schematically in Figure 1A. Mice carrying targeted alleles were genotyped by PCR using Taqman primers from Sigma (St. Louis, MO, USA) and GoTaq polymerase from Promega (Madison, WI, USA).

For *Rb1*, the 5′ and 3′ primers were SXV01, 5′-GAAAGGAAAGTCAGGGACATTGGG-3′; SXV02, 5′CTCAAGAGCTCAGACTCATGG; and SXV03, 5′-GGCGTGTGCCATCAATG-3′. The cycling conditions were 5 minutes as 95°C, 33 cycles of 30 seconds at 95°C/30 seconds at 58°C/50 seconds at 72°C, followed by 7 minutes at 72°C. The wild-type or floxed *Rb1* alleles resulted in 235-bp or 283-bp bands, respectively.

For *Tp53*, the 5′ and 3′ primers were CK339, 5′-GTTAAACCCAGCTTGACCAAGTG-3′; CK340, 5′-CTTGGTCTTCAGGTAGCTATAGG-3′; and CK341, 5′-CATAGGAGGCAGAGACAGTTGG-3′. The cycling conditions were 5 minutes as 95°C, 30 cycles of 30 seconds at 95°C/20 seconds at 61°C/60 seconds at 72°C, followed by 7 minutes at 72°C. The wild-type or floxed *Tp53* alleles resulted in 288-bp or 370-bp bands, respectively.

For *Cre*, the 5′ and 3′ primers were CK168, 5′-ACGGCAAATTCAACGGCACAG-3′; CK169, 5′-TTGAAGTCGCAGGAGACAACCTG-3′; CK190, 5′-GGATTTCCGTCTCTGGTGTAGC-3′; and CK191, 5′-ACCATTGCCCCTGTTTCACTATC-3′. 5 minutes as 95°C, 30 cycles of 60 seconds at 95°C/60 seconds at 66°C/2 minutes at 72°C, followed by 7 minutes at 72°C. The wild-type or *Cre* alleles resulted in 703-bp or 350-bp bands, respectively.

Primary cell cultures

All human tissue samples were collected following informed consent for an IRB-approved biorepository at OHSU.

At time of surgery or at necropsy, a 0.25-1 cm^3 portion of the tumor was minced with a sterile scalpel and digested at 37°C for 4-6 hours in 0.5% collagenase in Neurobasal-A media (Gibco, Life Technologies, Grand Island, NY, USA). If a prolonged digest was required, the specimen was digested overnight at 4°C. Following digestion, the specimen was rinsed with Neurobasal-A media twice and then strained through a 70 µm filter. Half of this single cell suspension was then plated in tissue culture dishes with serum-free media (Neurobasal-A with 2% antibiotic/antimycotic (Gibco) and supplemented with 10 ng/mL PDGF -AA (Shenandoah, Warminster, PA, USA), 10 ng/mL PDGF-BB (Shenandoah), 20 ng/mL hFGF (Promega), 20 ng/mL hEGF (Promega), and 1x B-27 (Gibco)). The remaining cells were plated in RPMI with fetal bovine serum (RPMI 1640 with 10% FBS, 2% antibiotic/antimycotic (Gibco), 2% sodium pyruvate (Gibco), 1% L-glutamine (Gibco) and 2 µM HEPES (Gibco)). Cells were incubated at $37^{o}C$ with humidified 5% CO_2. Fresh media was added weekly and cells were passaged when > 70% confluent.

Cell culture OS17 kindly provided by Dr. Pete Houghton and grown in RPMI supplemented as above.

Clinical presentation and outcomes

The patient PCB439 was an 18-year-old young white woman who presented with pain and swelling in her distal femur. High-grade conventional osteosarcoma with partial telengiectatic change was confirmed on biopsy. She received standard chemotherapy with vincristine, doxorubicin, cisplatin and high-dose methotrexate per COG protocol AOST0331. Resection specimen had 75% necrosis. On end of therapy imaging, a new solitary 4 mm pulmonary nodule was identified and increased in size on short-interval repeat chest CT. She underwent thoracoscopic resection and pathology confirmed metastatic osteosarcoma. On continued surveillance imaging, she had no further evidence of disease for the

next two and a half years. When she developed rapidly progressive numbness and weakness in her upper extremity, a spine MRI demonstrated a large mass at T1 with cord compression but no other evidence of disease, including no pulmonary disease or additional bony disease. She underwent urgent debulking and post-operative radiation, but declined chemotherapy. Five months later, she developed back pain and weakness in her bilateral lower extremities and was found to have a metastatic lesion in T4 with cord compression. She again underwent surgical debulking (specimen "PCB439") with post-operative radiation and again declined chemotherapy. While receiving radiation, she developed a palpable neck mass and worsening neck pain; CT imaging confirmed diffuse tumor involvement of the cervical spine with intracranial extension. She transitioned to hospice and died three and a half years after diagnosis and less than five months after relapse.

The patient PCB429 is a 23-year-old Latino man with high-grade osteoblastic osteosarcoma of the distal femur (biopsy specimen "PCB429"). He received standard chemotherapy with vincristine, doxorubicin, cisplatin and high-dose methotrexate per COG protocol AOST0331. He experienced resolution of pain after 3 weeks of chemotherapy and the resection specimen had 95% necrosis. He has no evidence of disease recurrence 19 months after initial diagnosis.

The patient PCB326 is a multiracial 19-year-old woman with high-grade conventional type osteosarcoma of the distal femur. She received standard chemotherapy with vincristine, doxorubicin, cisplatin and high-dose methotrexate per COG protocol AOST0331. Primary cell culture "PCB326c" was established from the resection specimen. Her resection specimen had > 80% viable tumor cells and she developed bilateral pulmonary nodules prior to the end of chemotherapy. Pulmonary nodules stable 3 years after initial diagnosis.

The patient PCB509 is a 12-year-old Caucasian boy with high-grade conventional osteosarcoma with pleomorphic and osteoblastic foci, with metastatic disease in lungs and bones at initial diagnosis. His primary tumor was in the proximal tibia, which contained skip lesions. Primary cell culture "PCB509c" was established from a biopsy specimen. He underwent upfront amputation followed by standard chemotherapy with vincristine, doxorubicin, cisplatin and high-dose methotrexate per COG protocol AOST0331. He is alive 1 year after initial diagnosis.

The dog PET-10 was a five-year-old spayed female English Sheepdog with a high-grade telangiectatic osteosarcoma of the distal radius. She underwent amputation (specimen "PET-10") followed by carboplatin. New pulmonary metastatic disease was evident on chest x-ray after three cycles and treatment was changed to vinorelbine briefly, then toceranib, and finally experimental therapy with bortezomib. She was compassionately euthanized for progressive disease less than 4 months after diagnosis. Samples from lung and spine metastases ("PET-10lung" and "PET-10spine") were collected at time of necropsy.

The dog PET-20 was a seven-year-old spayed female Rottweiler with osteogenic osteosarcoma of the distal radius. She underwent amputation (specimen "PET-20") followed by carboplatin. She was compassionately euthanized for progressive disease 5 months after diagnosis.

The dog PET-24 was an eight-year-old neutered male Golden Retriever with osteosarcoma of the distal radius. He underwent amputation (specimen "PET-24") followed by carboplatin. He did very well until 20 months after diagnosis, when he was compassionately euthanized for progressive disease.

The dog PET-1 was described previously (1), but briefly, he was a seven-year-old neutered male Golden Retriever with a high-grade osteosarcoma of the proximal tibia. Specimen "PET-1" was from amputation. He received five cycles of adjuvant carboplatin and taurolidine, followed by maintenance dasatinib. He did very well until 30 months after diagnosis, when chest x-rays demonstrated diffuse metastatic disease and the owners requested compassionate euthanasia.

The dog PET-7 was a 6-year-old neutered male Labrador Retriever with osteosarcoma of the distal radius. He underwent amputation and five adjuvant cycles of carboplatin, followed by maintenance toceranib. He was compassionately euthanized for metastatic disease 14 months after diagnosis. Primary cell culture "PET-7c" was established from a metastatic lesion biopsied at time of necropsy.

The dog PET-27 was a 8-year-old neutered male Labrador Retriever with osteosarcoma of the distal radius. He underwent amputation and primary cell culture "PET-27c" was established from the amputation specimen. No additional clinical information is available.

Primary cell culture validation

When possible, allografts or xenografts were established to confirm tumorigenicity of primary cell cultures. Cultured cells were injected into immunodeficient SCID hairless outbred strain mice (SHO #474, Charles River, Wilmington, MA, USA). Unilateral gastrocnemius injury was induced with injection of 50 μL of cardiotoxin (2.5 μM) 24 hours prior to orthotopic injection of allograft cells. Primary early passage cells grown in suspension as sarcomaspheres in serum-free media (PET7c, PET27c, 61236LUc, 61323Lc) were collected from tissue culture plates once spheres reached 50–100 μm in diameter, centrifuged at 400 × g for 3 minutes and resuspended in PBS. There was no attempt to disrupt sarcomaphere structure. Cells in a volume of 100 μL PBS were injected into the peritibial space of the previously

injured gastrocnemius compartment. 1 × $10e^6$ OS17 and PCB151JAX cells were suspended in a volume of 50 µL PBS and injected into uninjured subcutaneous flank tissue. Tumors were measured in three dimensions with calipers and volume calculated by 4/3π*length*width*height. Animals were sacrificed once tumors reached 2 cm^3.

Alternatively, copy number variation (CNV) within the primary cell culture was used as a proxy for tumorigenicity. Hybridization targets were prepared from genomic DNA using the SNP 6.0 Core Reagent Kit (Affymetrix, Santa Clara, CA) according to manufacturer's recommendations. DNA isolated from human primary cell cultures and matched normal peripheral blood or fresh frozen tissue was hybridized to a Genome-Wide Human SNP Array 6.0 (Affymetrix) and scanned following standard protocols. Image processing was performed using the Affymetrix GeneChip Command Console (AGCC) v. 3.1.1. GISTIC 2.0 analysis identified genes with somatic copy-number alterations. For each sample, each gene was considered a high-level deletion (-2), a low-level deletion (-1), a low level amplification (1), or a high-level amplification (2). A high deletion (or amplification) aberration score indicates the gene contains significantly more deletions (or additions), while a low deletion (or amplification) aberration score indicates only slightly more deletions (or additions). An aberration score of zero (0) indicates genes with no discernable copy number aberration.

Drug screening protocol

Each drug or PKIS compound was plated in four concentrations (10 µM, 1 µM, 100 nM, 10 nM) in triplicate in 384-well format. Cells were plated at a density of 2,500 cells per well and incubated at 37°C with humidified 5% CO_2 for three days. Cell viability was determined by CellTiterGlo luminescent assay (Promega #G7572) per manufacturer's instructions and measured with the IVIS Lumina II imaging system. Data was analyzed using GraphPad Prism software and results interpreted following curve fit by four-parameter nonlinear regression. Combination indexes were determined by CompuSyn v1.0.

Immunoblotting

Cultured cells or tumor tissue was lysed in radioimmunoprecipitation assay buffer containing both protease and phosphatase inhibitor (Sigma). Standard immunoblotting procedures were performed and proteins visualized with chemiluminescent signal using Super Signal Western Pico or Dura Substrate (Pierce Biotechnology). Immunoblot analysis used anti-ARK5 (Santa Cruz Biotechnology, sc-271828 and sc-130117) and anti-β-actin (Sigma, A1978).

siRNA

Cells were grown in antibiotic-free RPMI medium and 80% confluent at time of transfection. We utilized DharmaFECT 1 Transfection Reagent (Dharmacon, #T-2001) in Opti-MEM medium (ThermoFisher Scientific, #31985-070) at a final concentration of 2% with 50 nM of two targeted siRNAs (ON-TARGETplus Nuak1 siRNA, Dharmacon, #J-063024-05 and #J-063024-07) or 100 nM of non-targeting control siRNA (siGENOME Non-Targeting siRNA Pool #2, Dharmacon, #D-001206-14). Cells were lysed in radioimmunoprecipitation assay buffer with Halt protease and phosphatase inhibitor cocktail (ThermoFisher Scientific, #78440).

Patient-derived xenografts (PDX)

A PDX model from resection specimen PCB151 was established by The Jackson Laboratory per institutional protocol as part of the Primary Human Tumor Consortium. Fresh surgical tissue was implanted into NSG mice (stock number 005557) and approximately six months later, the tumor (JAX model TM00039) was expanded as P1. A portion of this fresh explant tissue ("PCB151JAX") was then returned to our laboratory, where we established a primary cell culture as described above.

REFERENCES

1. Davis LE, Hofmann NE, Li G, Huang ET, Loriaux MM, Bracha S, Helfand SC, Mata JE, Marley K, Mansoor A, Tyner JW, Abraham J, Séguin B, Keller C. A case study of personalized therapy for osteosarcoma. Pediatr Blood Cancer. 2013; 60:1313–9.

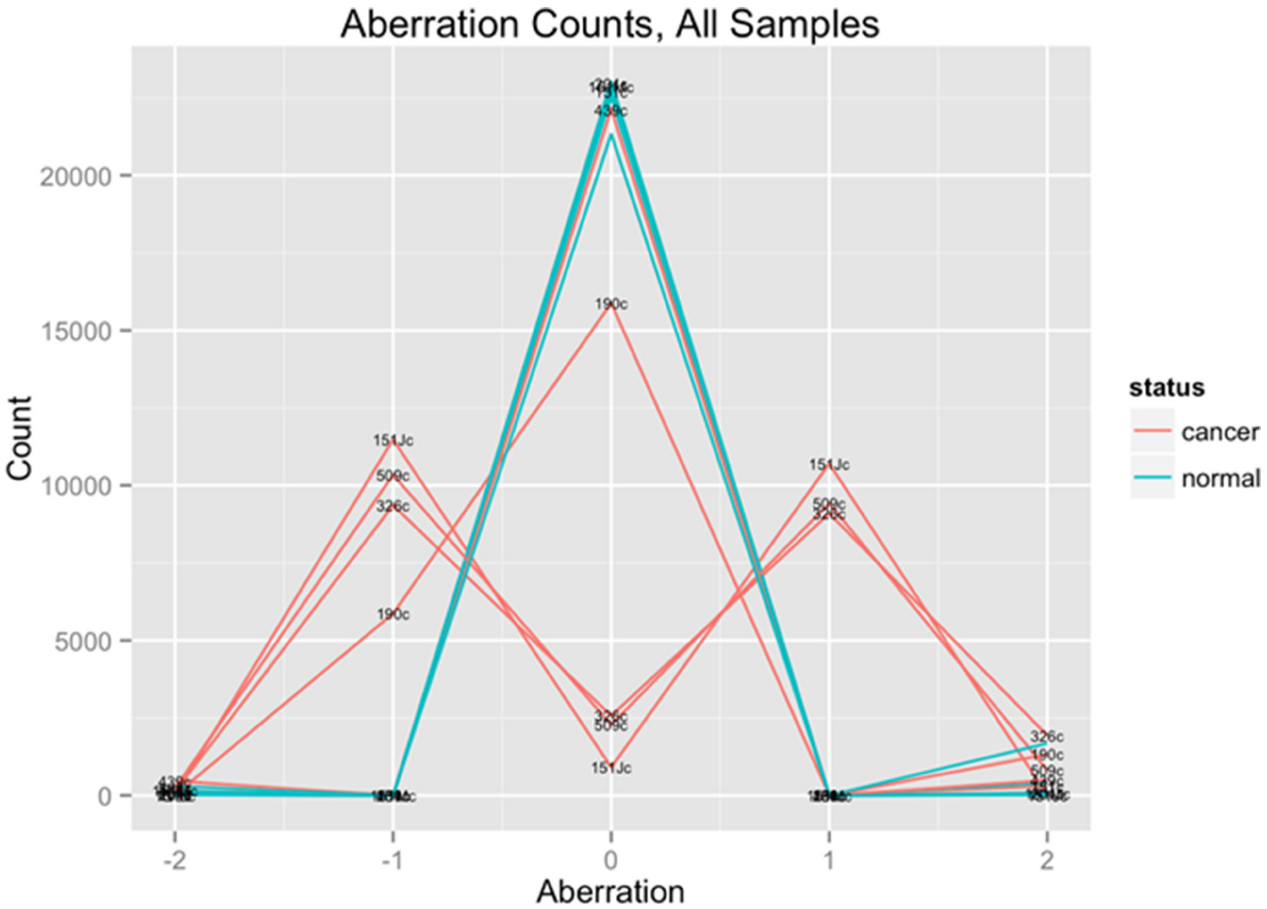

Supplementary Figure 1: Aberration call rates from GISTIC 2.0 analysis of human primary cell cultures. Three "clusters" are apparent: one with a distribution similar to normal samples, one distinct from the normal sample call rate distribution (highly aberrant and consistent with cancer), and a third indeterminate group with a call rate distribution distinct from both the normal and cancer call rate distributions, likely representing a mixed population of cells. 151Jc = PCB151JAX.

Karyotype:
65~69<3n>,XX,der(X)t(X;20)(q21.2;q11.2)del(X)(p11.2p11.4),del(2)(q35q35),-3,dup(6)(p12p21.3),+7,der(7)(7qter→7q22::7q11→7p11::7q22→7qter),-9, del(9)(p12),t(9;9)(p21;q22),der(10)t(3;10)(p21.3;q23.2),-13,-14,add(17)(p11.1), der(17)(17pter→17q11.2::?::3q21.3→qter),+18,add(18)(q21.1),der(18), t(1;18)(p31.3;q23),-20,add(21)(p11.2),der(21)t(1;21)(q21;p11.2), add(22)(q11.2),+mar1[12]/67~68,sl, +mar2[8]

Interpretation and Comments:
All twenty metaphase cells examined comprised two related, near-triploid, complex abnormal clones. The structural and numerical aberrations are delineated above relative to the triploid karyotype <3n>. There were fourteen clonal structural abnormalities observed in all of the cells, in addition to 7 numerical abnormalities. Two different markers of unknown chromosomal origin were also present, one observed in every cell and the other observed in eight of twenty cells.

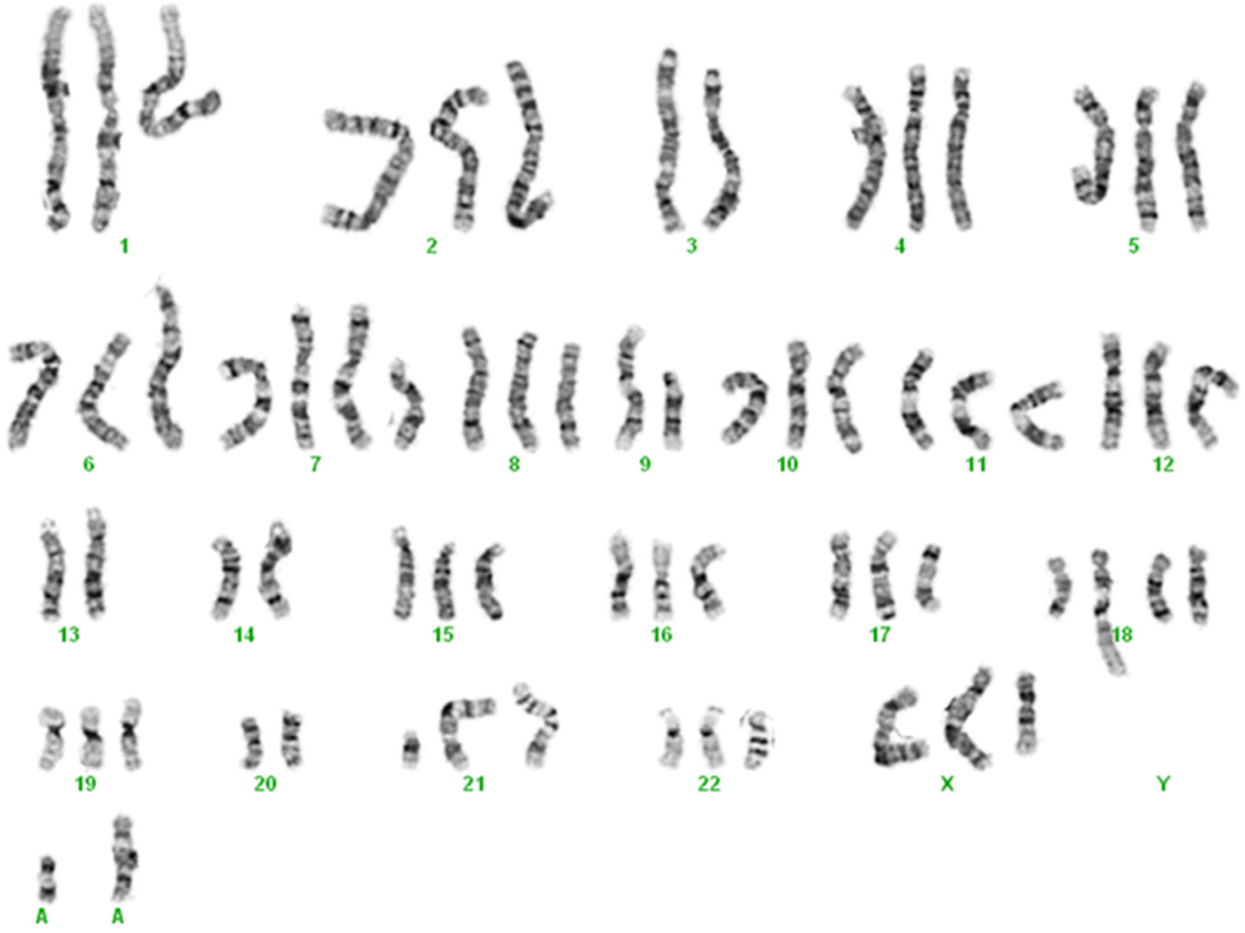

Supplementary Figure 2: Metaphase karyotype results for PCB151JAX cell culture.

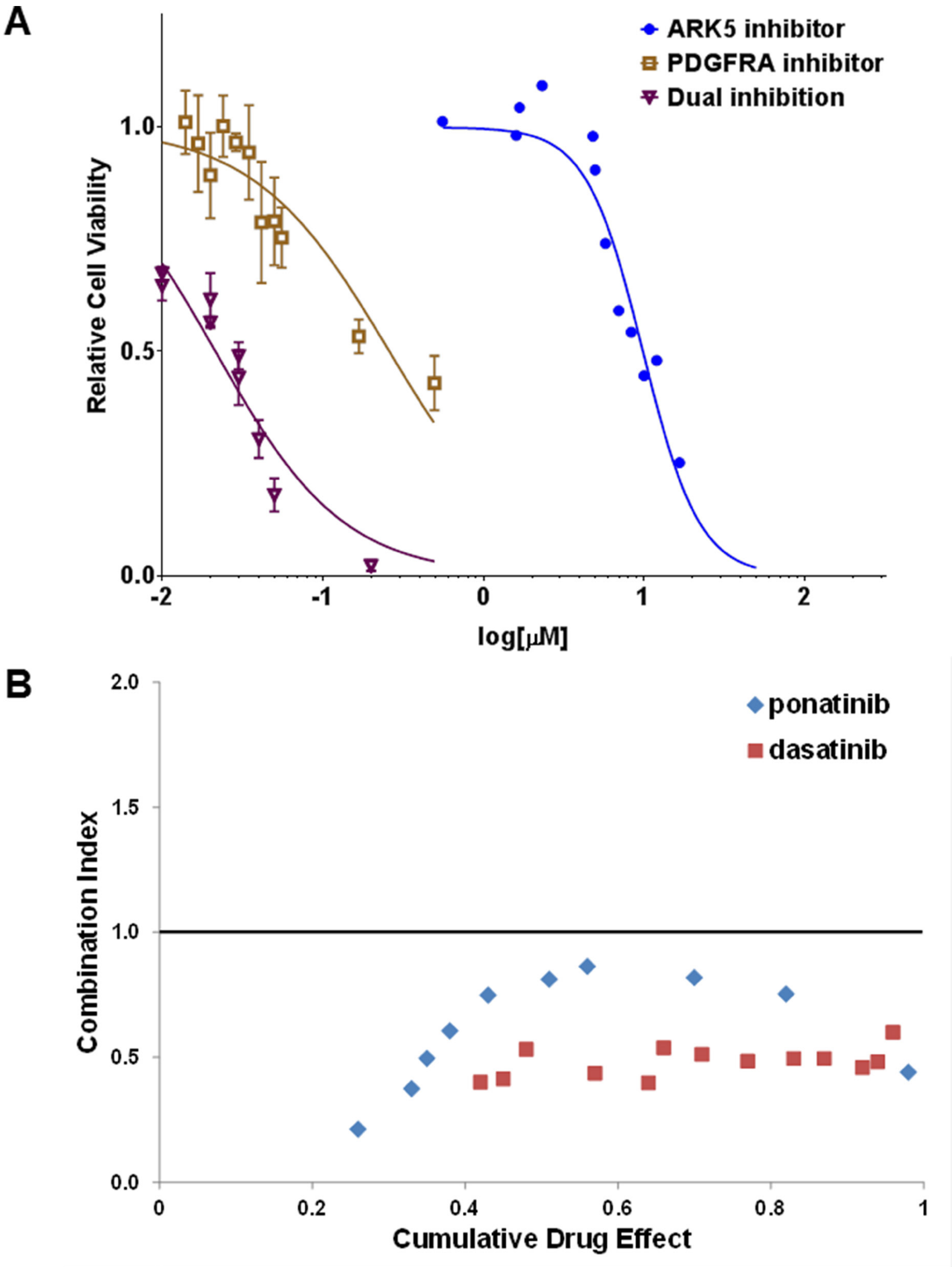

Supplementary Figure 3: ARK5 inhibitor HTH-01-015 is highly synergistic when combined with a PDGFRα inhibitor as determined by *in vitro* cell viability assays with OS GEM 61323Lc cells. (**A**) HTH-01-015 monotherapy is ineffective (IC_{50} 9.8 μM), but the addition of HTH-01-015 (ARK5 inhibitor) to ponatinib (PDGFRα inhibitor) shifts *in vitro* IC_{50} from 260 nM to 21 nM. (**B**) Simultaneous inhibition of ARK5 (with HTH-01-015) and PDGFRα (with either ponatinib or dasatinib) results in strong synergy as determined by Chou-Talalay method (combination index range 0.21–0.86).

Supplementary Table 1: Investigator-selected compounds included in functional drug screens. See Supplementary_Table_1

Supplementary Table 2: OS GEM mice with tumors

UID	Genotype	Age at sacrifice (days)	Volume of single largest tumor at sacrifice (cm^3)	Tumor site(s)	Cell Cultures Established
U61236	*Osx1-Cre, Trp53–/–, Rb1–/–*	263	1.87	hind limb; metastases to lungs, liver, lymph nodes	Yes, multiple – limb; liver; lung
U61319	*Osx1-Cre, Trp53–/–, Rb1–/–*	237	2.25	shoulder, hind limb	Yes, multiple – shoulder; limb
U61320	*Osx1-Cre, Trp53–/–, Rb1-/-*	138	< 0.25	face	Yes
U61322	*Osx1-Cre, Trp53–/–, Rb1–/–*	202	< 0.25	face	Yes
U61323	*Osx1-Cre, Trp53–/–, Rb1–/–*	262	2.73	bilateral hind limbs; metastases to lungs	Yes, multiple – left limb; right limb; lung
U61756	*Osx1-Cre, Trp53–/+, Rb1–/–*	409	1.55	bilateral hind limbs	No
U63244	*Osx1-Cre, Trp53–/+, Rb1–/–*	288	1.39	pelvis	No

Supplementary Table 3: List of mutated genes. See Supplementary_Table_3

Supplementary Table 4A: Human. See Supplementary_Table_4A

Supplementary Table 4B: Mouse. See Supplementary_Table_4B

Supplementary Table 4C: Canine. See Supplementary_Table_4C

Supplementary Table 5: Prioritized list (by unadjusted *p*-value) of significant pathways for patient PCB151 (top table) and OS GEM U61323L (bottom table). See Supplementary_Table_5
