# Supplementary material for: Integration of genomic, transcriptomic and functional profiles of aggressive osteosarcomas across multiple species
Source: Oncotarget. 2017 Jul 25;8(44):76241–56. doi: 10.18632/oncotarget.19532 (PMC5652702; doi:10.18632/oncotarget.19532)
Supplement: Supplementary file 2 [file oncotarget-08-76241-s002.docx]

| **Supplemental Table S1.** Investigator-selected compounds included in functional drug screens. | | |
| --- | --- | --- |
|  |  |  |
| **Drug/compound** | **target(s)** | ***synonyms*** |
| ABT-263 | BCL |  |
| ABT-737 | BCL2 |  |
| alisertib | Aurora kinase A | *MLN8237* |
| APR-246 | p53 | *PRIMA-1* |
| AUY922 | Hsp90 |  |
| AZD5363 | AKT |  |
| AZD8931 | pan-ERB |  |
| barasertib | Aurora kinase B | *AZD1152-HQPA* |
| BAY-11-7082 | NFkB |  |
| BEZ235 | PI3K, mTOR |  |
| BIO | GSK3β |  |
| BIX 01294 | methyltransferase |  |
| BMS 754807 | IGF1R |  |
| bortezomib | proteasome |  |
| cabozantinib | EGFR, c-MET | *XL-184* |
| carfilzomib | proteasome |  |
| CDK 1/2 inhibitor | CDK 1/2 |  |
| cediranib | VEGFR | *AZD2171* |
| celecoxib | COX2 |  |
| crizotinib | ALK, MET |  |
| CRM1 inhibitor | CRM1 |  |
| CX-4945 | CK2 |  |
| dasatinib | BCR-ABL, SRC |  |
| decitabine | DNA-methyltransferase | *5-Aza-2 deoxycytidine* |
| defactinib | FAK | *PF04554878* |
| dinaciclib | CDKs | *SCH-727965* |
| EM1421 | survivin |  |
| entinostat | HDAC 1/3 | *SNDX-275, MS-275* |
| enzastaurin | PKC-B | *LY317615* |
| eribulin | cytotoxic |  |
| fenretinide | retinoid | *4-HPR* |
| flavopiridol | CDKs | *alvocidib* |
| fluvastatin | HMGA1 |  |
| forskolin |  |  |
| fostamatinib | SYK | *R788* |
| ganciclovir | antiviral |  |
| GANT61 | Gli1 (SHH) |  |
| GSK126 | EZH2 |  |
| GSK-J4 | H3K27 demethylases |  |
| hydroxyglutaric acid | histone lysine demethylases |  |
| INK128 | TORC 1/2 |  |
| JIB-04 | KDM4 |  |
| JQ1 | bromodomain |  |
| lapatinib | EGFR |  |
| LCL161 | SMAC |  |
| lenalidomide | immune modulation |  |
| LGK974 | Wnt |  |
| linsitinib | IGF1R | *OSI-906* |
| LSD1 inhibitor | LSD1 |  |
| LY2090314 | GSK3β |  |
| LY2228820 | p38 MAPK |  |
| LY2606368 | Chk1 |  |
| LY2874455 | FGFR1-4 |  |
| LY293111 | Leukotriene B4 |  |
| LY2940680 | Smo |  |
| maraviroc | CCR5 |  |
| metformin | AMPKA |  |
| midostaurin | pan-kinase | *PKC412* |
| mithramycin | SP1-4 |  |
| MK1775 | WEE1 |  |
| MK2206 | AKT |  |
| N-acetylcysteine | free radicals | *NAC* |
| netropsin | HMGA2 |  |
| nilotinib | PDGF-R, c-Kit, BCR-ABL | *AMN-107* |
| obatoclax | BCL2 | *GX15-070* |
| palbociclib | CDK 4/6 | *PD0332991* |
| panobinostat | HDAC | *LBH-589* |
| pazopanib | VEGFRs, c-KIT, PDGFR |  |
| pelitinib | EGFR | *EKB-569* |
| pemetrexed | cytotoxic |  |
| PF573228 | FAK |  |
| ponatinib | BCR-ABL, VEGFR2, FGFR1, PDGFRα | *AP24534* |
| quinacrine | unknown |  |
| RO4929097 | Notch |  |
| RO5045337 | MDM2 | *nutlin* |
| ruxolitinib | JAK 1/2 | *INCB018424* |
| saracatinib | SRC | *AZD0530* |
| SB431542 | TGFβ |  |
| selumetinib | MEK1/2 inhibitor | *AZD6244* |
| SGC0946 | DOT1L |  |
| SGI-1776 | PIM kinase |  |
| sirolimus | mTOR | *rapamycin* |
| SJ-172550 | MDMX |  |
| SNS-032 | CDK 2/7/9 | *BMS 387032* |
| sodium butyrate | HDAC |  |
| sorafenib | VEGFR, PDGFR, RAF |  |
| SP600125 | JNK 1/2/3 |  |
| tamoxifen | ER |  |
| temsirolimus | mTOR |  |
| thapsigargin | SERCA |  |
| tozasertib | Aurora kinase A/B/C | *MK-0457, VX-680* |
| trametinib | MEK |  |
| tretinoin | differentiation agent | *all-trans retinoic acid, ATRA* |
| trichostatin | HDAC |  |
| tubastatin | HDAC 6 |  |
| vandetanib | VEGFR-2, EGFR, RET | *ZD6474* |
| veliparib | PARP-1 and PARP-2 | *ABT-888* |
| vemurafenib | BRAF | *PLX4032* |
| VER-155008 | Hsp90, Hsp70 |  |
| vismodegib | Smo (SHH) | *GDC-0449* |
| volasertib | PLK1 |  |
| vorinostat | HDAC | *SAHA* |
| YM155 | survivin |  |
| zebularine | DNA methylation |  |
| zibotentan | endothelin | *ZD4054* |
