# Supplementary material for: Integration of genomic, transcriptomic and functional profiles of aggressive osteosarcomas across multiple species
Source: Oncotarget. 2017 Jul 25;8(44):76241–56. doi: 10.18632/oncotarget.19532 (PMC5652702; doi:10.18632/oncotarget.19532)
Supplement: Supplementary file 5 [file oncotarget-08-76241-s005.docx]

| **Supplementary Table S5.**  Prioritized list (by unadjusted *p*-value) of significant pathways for  PCB151 (top table) and U61323L (bottom table). | | | | | | | |
| --- | --- | --- | --- | --- | --- | --- | --- |
|  |  |  |  |  |  |  |  |
| **Pathways with significant DNA & RNA aberrations in PCB151 (human)** | **Pathway p-value** | **Pathway Size (# genes)** | **Pathway adjusted p-value** | **Number of DE Genes** | **Number of Mutated Genes** | **Number of Target Genes** |  |
| Regulation of PLK1 Activity at G2/M Transition | 0.004 | 78 | 1 | 6 | 1 | 3 |  |
| Loss of Nlp from mitotic centrosomes | 0.004 | 60 | 1 | 5 | 1 | 2 |  |
| Loss of proteins required for interphase microtubule organization from the centrosome | 0.004 | 60 | 1 | 5 | 1 | 2 |  |
| Phospholipase C-mediated cascade; FGFR2 | 0.006 | 47 | 1 | 5 | 0 | 1 |  |
| Extracellular matrix organization | 0.008 | 258 | 1 | 13 | 1 | 1 |  |
| Transcriptional regulation of white adipocyte differentiation | 0.013 | 75 | 1 | 6 | 0 | 1 |  |
| Centrosome maturation | 0.013 | 78 | 1 | 5 | 1 | 2 |  |
| Recruitment of mitotic centrosome proteins and complexes | 0.013 | 78 | 1 | 5 | 1 | 2 |  |
| Developmental Biology | 0.016 | 503 | 1 | 19 | 3 | 7 |  |
| Sema4D mediated inhibition of cell attachment and migration | 0.016 | 8 | 1 | 2 | 0 | 1 |  |
| Platelet activation, signaling and aggregation | 0.017 | 217 | 1 | 10 | 1 | 2 |  |
| FGFR2b ligand binding and activation | 0.02 | 10 | 1 | 2 | 0 | 1 |  |
| Anchoring of the basal body to the plasma membrane | 0.021 | 85 | 1 | 5 | 1 | 2 |  |
| Activation of the AP-1 family of transcription factors | 0.021 | 10 | 1 | 2 | 0 | 1 |  |
| Phospholipase C-mediated cascade; FGFR3 | 0.024 | 42 | 1 | 4 | 0 | 1 |  |
| Negative regulation of FGFR2 signaling | 0.025 | 28 | 1 | 3 | 0 | 1 |  |
| Phospholipase C-mediated cascade; FGFR4 | 0.028 | 45 | 1 | 4 | 0 | 1 |  |
| FRS-mediated FGFR1 signaling | 0.03 | 28 | 1 | 3 | 0 | 1 |  |
| G2/M Transition | 0.031 | 120 | 1 | 6 | 1 | 6 |  |
| Mitotic G2-G2/M phases | 0.033 | 122 | 1 | 6 | 1 | 6 |  |
| Oxidative Stress Induced Senescence | 0.035 | 117 | 1 | 5 | 1 | 2 |  |
| Activated point mutants of FGFR2 | 0.036 | 14 | 1 | 2 | 0 | 1 |  |
| FGFR2 mutant receptor activation | 0.036 | 14 | 1 | 2 | 0 | 1 |  |
| repression of WNT target genes | 0.038 | 13 | 1 | 2 | 0 | 1 |  |
| FRS-mediated FGFR2 signaling | 0.041 | 32 | 1 | 3 | 0 | 2 |  |
| Downstream signal transduction | 0.047 | 150 | 1 | 8 | 0 | 7 |  |
| Signaling by PDGF | 0.049 | 176 | 1 | 9 | 0 | 7 |  |
| FGFR2 ligand binding and activation | 0.05 | 17 | 1 | 2 | 0 | 1 |  |
|  |  |  |  |  |  |  |  |
| **Pathways with significant DNA & RNA aberrations in U61323L (mouse)** | **Pathway p-value** | **Pathway Size (# genes)** | **Pathway adjusted p-value** | **Number of DE Genes** | **Number of Mutated Genes** | **Number of Target Genes** |  |
| Glucose metabolism | <0.001 | 73 | 0.09 | 11 | 1 | 1 |  |
| Extracellular matrix organization | 0.001 | 238 | 0.71 | 18 | 3 | 4 |  |
| Hemostasis | 0.001 | 465 | 0.71 | 26 | 7 | 28 |  |
| Meiotic Recombination | 0.002 | 80 | 1 | 2 | 6 | 2 |  |
| NoRC negatively regulates rRNA expression | 0.002 | 82 | 1 | 2 | 6 | 2 |  |
| Response to elevated platelet cytosolic Ca2+ | 0.002 | 85 | 1 | 9 | 1 | 2 |  |
| Cell-Cell communication | 0.004 | 103 | 1 | 9 | 2 | 7 |  |
| Negative epigenetic regulation of rRNA expression | 0.005 | 90 | 1 | 2 | 6 | 2 |  |
| Meiosis | 0.005 | 105 | 1 | 3 | 6 | 2 |  |
| Platelet activation, signaling and aggregation | 0.009 | 202 | 1 | 13 | 3 | 17 |  |
| Integrin cell surface interactions | 0.009 | 69 | 1 | 7 | 1 | 1 |  |
| Cell junction organization | 0.011 | 59 | 1 | 5 | 2 | 1 |  |
| Epigenetic regulation of gene expression | 0.016 | 104 | 1 | 2 | 6 | 2 |  |
| FGFR3b ligand binding and activation | 0.020 | 6 | 1 | 1 | 1 | 1 |  |
| G alpha (q) signalling events | 0.022 | 159 | 1 | 11 | 1 | 1 |  |
| Stimuli-sensing channels | 0.028 | 87 | 1 | 4 | 4 | 3 |  |
| Metabolism of carbohydrates | 0.044 | 243 | 1 | 14 | 2 | 1 |  |
